# A key role for sex chromosomes in the regulation of parthenogenesis in the brown alga *Ectocarpus*

**DOI:** 10.1371/journal.pgen.1008211

**Published:** 2019-06-13

**Authors:** Laure Mignerot, Komlan Avia, Remy Luthringer, Agnieszka P. Lipinska, Akira F. Peters, J. Mark Cock, Susana M. Coelho

**Affiliations:** 1 Sorbonne Université, UPMC Univ Paris 06, CNRS, Algal Genetics Group, UMR 8227, Integrative Biology of Marine Models, Station Biologique de Roscoff, Roscoff, France; 2 Bezhin Rosko, Santec, France; University of Cologne, GERMANY

## Abstract

Although evolutionary transitions from sexual to asexual reproduction are frequent in eukaryotes, the genetic bases of these shifts remain largely elusive. Here, we used classic quantitative trait analysis, combined with genomic and transcriptomic information to dissect the genetic basis of asexual, parthenogenetic reproduction in the brown alga *Ectocarpus*. We found that parthenogenesis is controlled by the sex locus, together with two additional autosomal loci, highlighting the key role of the sex chromosome as a major regulator of asexual reproduction. We identify several negative effects of parthenogenesis on male fitness, and different fitness effects of parthenogenetic capacity depending on the life cycle generation. Although allele frequencies in natural populations are currently unknown, we discuss the possibility that parthenogenesis may be under both sex-specific selection and generation/ploidally-antagonistic selection, and/or that the action of fluctuating selection on this trait may contribute to the maintenance of polymorphisms in populations. Importantly, our data provide the first empirical illustration, to our knowledge, of a trade-off between the haploid and diploid stages of the life cycle, where distinct parthenogenesis alleles have opposing effects on sexual and asexual reproduction and may help maintain genetic variation. These types of fitness trade-offs have profound evolutionary implications in natural populations and may structure life history evolution in organisms with haploid-diploid life cycles.

## Author summary

Asexual reproduction is widespread among all major clades of eukaryotes. Parthenogenesis represents a specific mode of asexual reproduction, secondarily derived from sexual reproduction, and refers to the development of a multicellular organism from an unfertilised gamete. Parthenogenesis has evolved independently in a wide variety of groups, but the genetic basis and the evolutionary forces driving transitions from sexual to parthenogenetic reproduction remain elusive. Here, we explore genetic, genomic and transcriptomic data from the brown alga *Ectocarpus* to uncover the genetic architecture of parthenogenesis. The brown algae are a group of complex multicellular organisms that have been evolving independently from animals and plants for more than a billion years, and they have recently emerged as important models to study the evolution of reproductive modes. We show that parthenogenesis is a complex genetic trait under the control of the sex locus, together with two additional autosomal quantitative trait loci, highlighting the critical role for the sex chromosomes in the control of asexual reproduction in this organism. We identify several negative effects of parthenogenesis on male fitness and reveal evidence for trade-offs between sexual and asexual reproduction during the life cycle of *Ectocarpus*. Our results support the idea that parthenogenesis may be under both sex-specific selection and generation/ploidally-antagonistic selection, but the action of fluctuating selection on this trait may also contribute to the maintenance of polymorphisms in populations.

## Introduction

Although sexual reproduction, involving meiosis and gamete fusion, is almost ubiquitous across eukaryotes, transitions to asexual reproduction have arisen remarkably frequently [1]. These asexual reproductive mechanisms include parthenogenesis, which involves the development of an embryo from an unfertilized gamete [1]. The genetic basis of parthenogenesis remains largely elusive in both plants and animals, although the factors triggering the transition to asexual reproduction have been most intensively studied in plants, motivated by the potential use of asexual multiplication in the production of agricultural crops (e.g.[2,3]). In plants, parthenogenesis is a component of apomixis, which is the asexual formation of seeds, resulting in progeny that are genetically identical to the mother plant. In gametophytic apomixis, the embryo sac develops either from a megaspore mother cell without a reduction in ploidy (diplospory) or from a nearby nucellar cell (apospory) in a process termed apomeiosis. Apomeiosis is then followed by parthenogenesis, which leads to the development of the diploid egg cell into an embryo, in the absence of fertilization (reviewed in [4]).

In some apomictic plants, inheritance of parthenogenesis is strictly linked to an apomeiosis locus (reviewed in [5]), whereas in other species the parthenogenesis locus segregates independently of apomeiosis [6–8]. For example, apomixis in *Hieracium* is controlled by two loci termed *LOSS OF APOMEIOSIS* (*LOA*) and *LOSS OF PARTHENOGENESIS* (*LOP*), involved in apomeiosis and parthenogenesis, respectively [9]. A third locus (*AutE*) involved in autonomous endosperm formation, was shown to be tightly linked to the LOP locus [10]. In *Pennisetum squamulatum*, apomixis segregates as a single dominant locus, the apospory-specific genomic region (ASGR), and recent work has highlighted a role for PsASGR-BABY BOOM-like, a member of the BBM-like subgroup of APETALA 2 transcription factors residing in the ASGR, in controlling parthenogenesis [11].

There has been very little interest in parthenogenesis in organisms outside the animal and plant lineages, despite the fact that this mode of reproduction is widespread across the eukaryotic tree [12–17]. These alternative systems could provide novel insights into the genetic basis of parthenogenesis. For example, parthenogenesis is a common phenomenon in the brown algae, a group of multicellular eukaryotes that has been evolving independently from animals and plants for more than a billion years [18], and can be an integral component of a species life cycle ([19–21]). Once released into the surrounding seawater, gametes of brown algae may fuse with a gamete of the opposite sex, to produce a zygote which will develop into a diploid heterozygous sporophyte. Alternatively, in some brown algae, gametes that do not find a partner will develop parthenogenically, as haploid (partheno-)sporophytes (e.g. [22]). Parthenogenesis in brown algae can therefore be equated with gametophytic embryogenesis in plants, where embryos are produced from gametes [23], but in the case of brown algae the parthenogenetic gamete is haploid. The brown algae are therefore excellent models to study the molecular basis of parthenogenesis because gametes are produced directly by mitosis from the multicellular haploid gametophyte, allowing parthenogenesis to be disentangled from apomeiosis. Although parthenogenesis has been described in several species of brown algae (e.g.[19,24,25]), the genetic basis, the underlying mechanisms and the evolutionary drivers and consequences of this process remain obscure.

**Fig 1:**
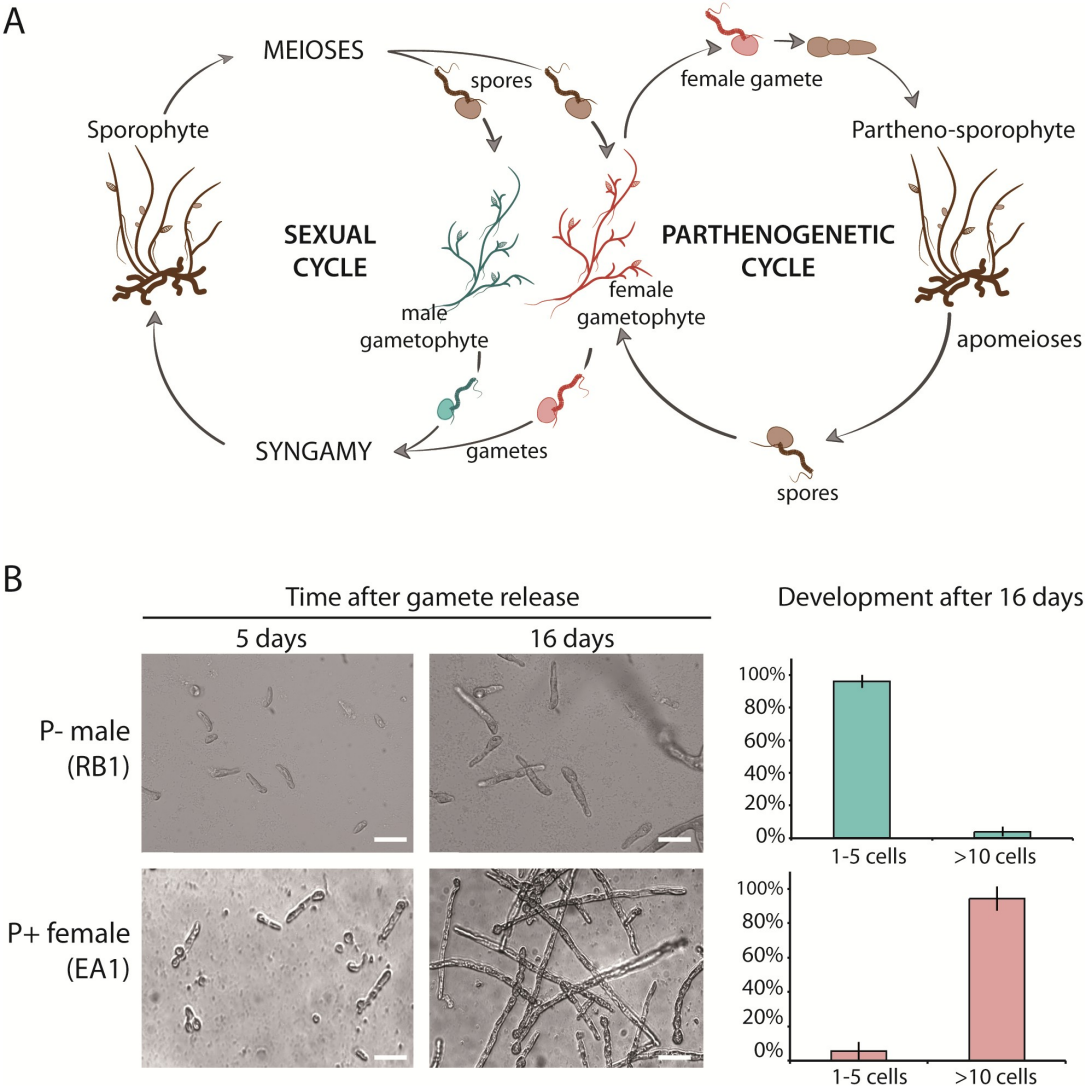
Life cycle of *Ectocarpus siliculosus* and phenotypes of parthenogenetic and non-parthenogenetic strains. **A**. Schematic representation of the life cycle of *Ectocarpus siliculosus*. *E*. *siliculosus* alternates between a gametophyte (haploid) and sporophyte (diploid) generation. Meiosis is carried out in unilocular sporangia on the sporophyte, producing male and female meio-spores. Meio-spores develop by mitosis into male or female gametophytes, which at maturity produce male or female gametes. Syngamy reconstitutes the diploid genome. The parthenogenetic cycle involves parthenogenesis of a gamete when it fails to encounter a gamete of the opposite sex. The parthenogenetic cycle can be completed either via an apomeiosis to produce meio-spores from a haploid partheno-sporophyte or via endoreduplication during partheno-sporophyte development, allowing meiosis to occur [19]. **B**. Photographs of the parthenogenetic growth of gametes of non-parthenogenetic male (RB1, top) and parthenogenetic female (EA1, bottom) strains of *Ectocarpus siliculosus* after 5 days and 16 days of development. Scale bar = 25 μm. The right panel shows the percentage of 1–5 cell and >10 cell partheno-sporophytes after 16 days of development for P- male gametes (strains Ec08, Ec398, Ec400, Ec409, Ec414, n = 2632) and P+ female gametes (strains Ec399, Ec402, Ec404, Ec406, Ec410, Ec412, Ec415, n = 3950).

The haploid-diploid life cycle of the model brown alga *Ectocarpus* involves an alternation between a haploid gametophyte and a diploid sporophyte. Superimposed on this sexual cycle, an asexual, parthenogenetic cycle has been described for some *Ectocarpus* strains (Fig 1A) [19,22]. In this parthenogenetic cycle, gametes that fail to meet a partner of the opposite sex develop into haploid partheno-sporophytes. These partheno-sporophytes are indistinguishable morphologically from diploid sporophytes [19]. Partheno-sporophytes can produce gametophyte progeny to return to the sexual cycle through two mechanisms: 1) endoreduplication during development to produce diploid cells that can undergo meiosis or 2) individuals that remain haploid can initiate apomeiosis [19,22].

Here, we used a quantitative trait locus (QTL) approach to investigate the genetic basis of parthenogenesis in *Ectocarpus siliculosus*. We show that parthenogenesis is a complex genetic trait under the control of three QTLs, one major QTL located on the sex chromosome, another on chromosome 18, and a further minor QTL also on chromosome 18. We used genomic and transcriptomic analyses to establish a list of 77 candidate genes within the QTL intervals. We detected significant sex-by-genotype interactions for parthenogenetic capacity, highlighting the critical role of the sex chromosome in the control of asexual reproduction. Moreover, we observed negative effects of parthenogenetic capacity on male fitness in sexual crosses indicating trade-offs between sexual and asexual reproduction during the life cycle of *Ectocarpus*. Antagonistic selection for different alleles at the same locus in males and females, or during the haploid and diploid life cycle phases, has been shown to be able to maintain stable genetic polymorphisms ([26,27]). Likewise, temporal or spatial habitat heterogeneity may cause strong fluctuating selection on sex-specific traits, thereby contributing to the maintenance of genetic variation in populations [28]. Based on our observations, we discuss the potential roles of sexually-antagonistic, ploidally-antagonistic and fluctuating selection in the maintenance of polymorphism at the parthenogenesis QTLs.

## Results

### Parthenogenesis is controlled genetically

To precisely quantify the parthenogenetic capacity of two strains of *E*. *siliculosus*, clonal cultures of male (RB1) and female (EA1) gametophytes, collected from a field population in Naples (S1 Table), were induced to release gametes under strong light (see methods) and pools of male and female gametes were allowed to settle separately, without mixing of the two sexes, on coverslips. Development of the gametes was then followed for 16 days (Fig 1B, S1 Table). After 5 days, both male and female gametes had started to germinate and went through the first cell divisions. After 16 days, 94% of the female gametes on average had grown into >10 cell filaments, whereas 96% of the male gametes remained at the 3–4 cell stage and cell death was observed after about 20 days. Strains were therefore scored as parthenogenetic if more than 4% of parthenotes grew beyond the ten-cell stage (Fig 1B).

In several brown algal species, unfused male and female gametes show different parthenogenetic capacities, and it is usually the female gametes that are capable of parthenogenesis whereas male gametes are non-parthenogenic (e.g. [25,29]). To investigate if there was a link between parthenogenetic capacity and sex, we crossed the female (EA1) P+ strain with the male (RB1) P- strain described above (S1 Fig, S1 Table). The diploid heterozygous zygote resulting from this cross (strain Ec236) was used to generate a segregating family of 272 haploid gametophytes. These 272 siblings were sexed using molecular markers [30] and their gametes phenotyped for parthenogenetic capacity (see above). The segregating population was composed of 144 females and 128 males, consistent with a 1:1 segregation pattern (chi2 test; p-value = 0.33, S2 Table). Phenotypic assessment of the parthenogenetic capacity of the gametes released by each gametophyte revealed a significant bias in the inheritance pattern, with 84 individuals presenting a P- phenotype and 188 a P+ phenotype (Chi2 test; p-value = 2.86x10^-10^) (S2 and S3 Tables). Strikingly, all female strains exhibited a P+ phenotype whereas, although most male stains exhibited a P- phenotype, 30% of males were P+ (S2 Table). This result indicated the presence of a parthenogenesis locus (or loci) that was not fully linked to the sex locus, and suggested a complex relationship between gender and parthenogenetic capacity.

### Stability of the parthenogenetic phenotype

A subset of the segregating family derived from the EA1 x RB1 cross was tested for phenotype stability. We cultivated two male P+ gametophytes, two male P- gametophytes and two female P+ gametophytes under different environmental conditions, varying light levels and temperature. After two weeks in culture, fertility was induced, and the parthenogenetic capacity of the gametes was scored (S4 Table). The parthenogenetic phenotype of all strains was stably maintained regardless of the culture conditions.

We also tested the stability of the parthenogenetic phenotype across generations: gametes of each of the three types (male P+, male P- and female P+) were allowed to develop into partheno-sporophytes. Note that this experiment is possible with P- males because a small proportion of male P- gametes (less than 4%) does not exhibit growth arrest and is able to grow to maturity. After two weeks in culture, gamete-derived partheno-sporophytes produced unilocular sporangia and released spores that developed into gametophytes. This second generation of gametophytes was again phenotyped for parthenogenetic capacity, and the results showed without exception that the parthenogenetic phenotype was stably maintained across generations (S4 Table).

To further investigate the inheritance of parthenogenetic capacity, a male P+ individual was crossed with a P+ female (S1 Fig). A total of 23 gametophyte lines were produced from two heterozygous sporophytes resulting from this cross. Phenotyping for sex and parthenogenesis revealed that all gametophyte lines exhibited a P+ phenotype, regardless of their sex (S5 Table). We concluded that parthenogenesis is controlled by (a) genetic factor(s).

### Generation of a genetic map for *E*. *siliculosus* and identification of the sex-determining region

**Fig 2:**
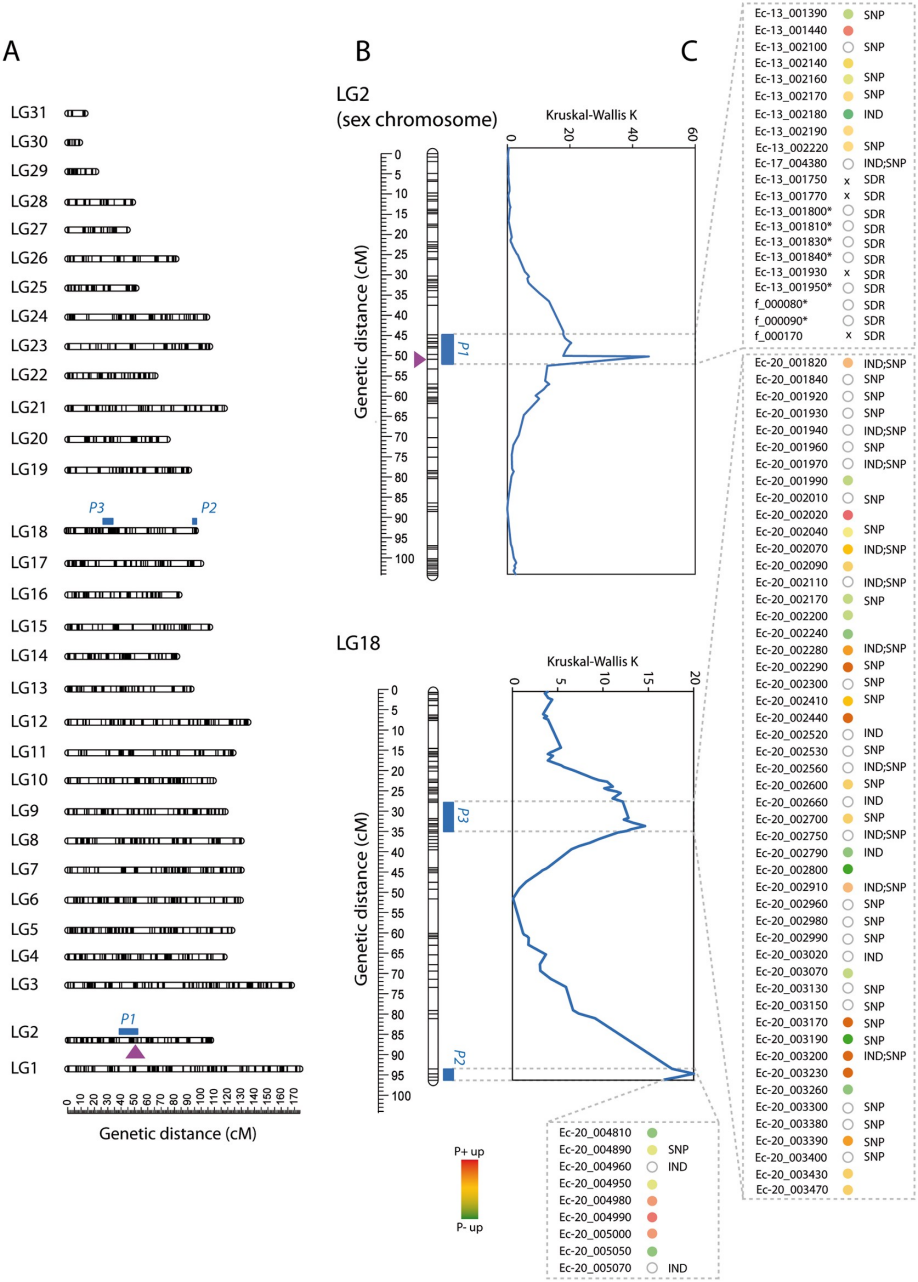
Quantitative trait loci identified for parthenogenetic capacity in *Ectocarpus siliculosus*. **A**. The 31 *Ectocarpus siliculosus* linkage groups showing the localization of QTLs for parthenogenesis. The position of the SDR is represented by a mauve arrow. **B**. QTL intervals were detected using the Kruskal Wallis test (blue). **C**. Candidate parthenogenesis genes in each QTL interval. Genes in QTL intervals were selected based on differential expression of their orthologs in P+ versus P- in gametes (colour dots) and polymorphisms in exons that are predicted to modify the protein product (IND and/or SNP). *SDR gametologue; x, sex-specific gene.

To produce a genetic map based on the EA1 x RB1 cross, a ddRAD-seq library was generated using 152 lines of the segregating progeny (S1 Fig) and sequenced on an Illumina HiSeq 2500 platform. A total of 595 million raw reads were obtained, of which 508 million reads passed the quality filters with a Q30 of 74.1%. A catalogue of 8648 SNP loci was generated using filtered reads from the parental strains and the STACKS pipeline (version 1.44) [31]. Twenty-eight individuals were removed due to excessive missing genotypes (see Methods) and highly distorted markers were also removed. The final map was constructed with 124 individuals and contained 5595 markers distributed across 31 linkage groups (LGs) and spanning 2947.5 centimorgans (cM). The average spacing between two adjacent markers was 0.5 cM and the largest gap was 17.6 cM (on LG23). The lengths of the 31 LGs ranged from 174 cM with 397 markers to 13 cM with 31 markers (Fig 2A, S6 Table). The 5595 markers included one non-SNP-based marker that was not derived from the RAD-seq library. This PCR-based marker was used to map the sex-determining region of the sex chromosome because the high level of divergence between male and female sequences in this region precludes the generation of co-dominant RAD-seq markers. This sex marker was mapped to position 51 cM on linkage group 2, thereby identifying this linkage group as the sex chromosome.

Note that the Peruvian *Ectocarpus* strain that was used to generate the reference genome sequence [32] was originally taxonomically classified as *Ectocarpus siliculosus* but subsequent analysis has demonstrated that this strain actually belongs to a distinct species within the *Ectocarpus siliculosi* group [33]. The genetic map generated here using *bona fide Ectocarpus siliculosus* strains is therefore for a novel species relative to the genetic maps generated for the Peruvian strain [34,35].

### QTL mapping approach to identify loci involved in parthenogenesis

To decipher the genetic architecture of parthenogenesis in *E*. *siliculosus*, we applied an “all-or-none” phenotyping and a QTL mapping approach [36,37], by considering P+ and P- as the two most ‘extreme’ phenotypes. We used the high-resolution genetic map to statistically associate markers with the P+ and P- phenotypes in the segregating family described above.

QTL mapping and association analysis identified three QTLs for parthenogenesis: two large-effect QTLs (r^2^ > 15%) and one smaller-effect QTL (r^2^ = 11.9%) (Fig 2A). Together, these three QTLs explained 44.8% of the phenotypic variance. The QTLs were located on two different LGs, LG2 and LG18 (Fig 2A). One of the large effect parthenogenesis QTLs (*P1*), which is located on LG2, co-localized with the sex marker, corresponding to the SDR of the sex chromosome (Fig 2). The *P1* locus was detected at the highest significance level (p-value <0.0001) with the Kruskal-Wallis statistical test (K* = 20.392). The other major effect locus, which we refer to as the *P2* locus, was located on LG18, and was also detected at the highest significance level with a Kruskal-Wallis statistical test (p-value<0.0001,K* = 19.993)(S7 Table). A non-parametric interval mapping (IM) method detected both the *P1* and *P2* loci, and indicated a proportion of variance explained (PVE) of 16.6% for *P1* and 16.3% for *P2*. The *P1* locus spanned 13.36 cM from 44.84 to 52.0 cM with a peak position at 51.0 cM whereas the *P2* locus spanned 2.82 cM, from 92.77 to 95.59 cM with a peak position at 93.98 cM.

The third QTL (*P3*), which was also located on LG18, was detected only with the Kruskal-Wallis statistical test (K* = 14.634, p-value<0.0005). The *P3* QTL had a smaller effect than *P1* and *P2*, and explained 11.9% of the phenotypic variance (Fig 2A and 2B; S7 Table).

Note that the QTL mapping described above was implemented using all 152 progeny (S1 Fig), which included both male and female strains. To investigate the contribution of the sex-specific, non-recombining region of the sex chromosome, we performed the same analysis using a subset of 93 male strains. The result showed that when females were excluded, the *P1* and the *P3* QTLs were not detected, and only the QTL located on LG18 (*P2*) was significantly detected (S7 Table). The absence of detection of the *P1* QTL was not a result of reduced statistical power due to the small sample size, because the QTL was detected when a sub-sample of 93 male and female individuals with the same sex ratio as the full 124 samples was used (S7 Table). The minor *P3* QTL was at the limit of significance when the 93 sub-sampled individuals were used, suggesting that the reduced sample size prevented the detection of this minor QTL.

To further characterise the cross between the two parthenogenetic strains Ec236-91 (female P+) x Ec236-202 (male P+) (S1 Fig), both strains were genotyped to determine which alleles of the autosomal parthenogenesis QTLs they carried. The maternal strain Ec236-91 carried the B (maternal) allele at the *P2* locus and the A (paternal) allele at the *P3* locus, whereas the male P+ strain Ec236-202 had B alleles at both the *P2* and *P3* loci (S8 Table). All the male progeny of this cross had a P+ phenotype (S5 Table), indicating that a B allele at the *P2* locus is sufficient to confer male gamete parthenogenesis.

### Epistasis analysis

An epistasis analysis was carried out to detect potential interactions between the parthenogenesis QTLs. Two analyses were performed, using either all 152 male and female progeny (‘full dataset’) or the subset of all the 93 male individuals.

**Fig 3:**
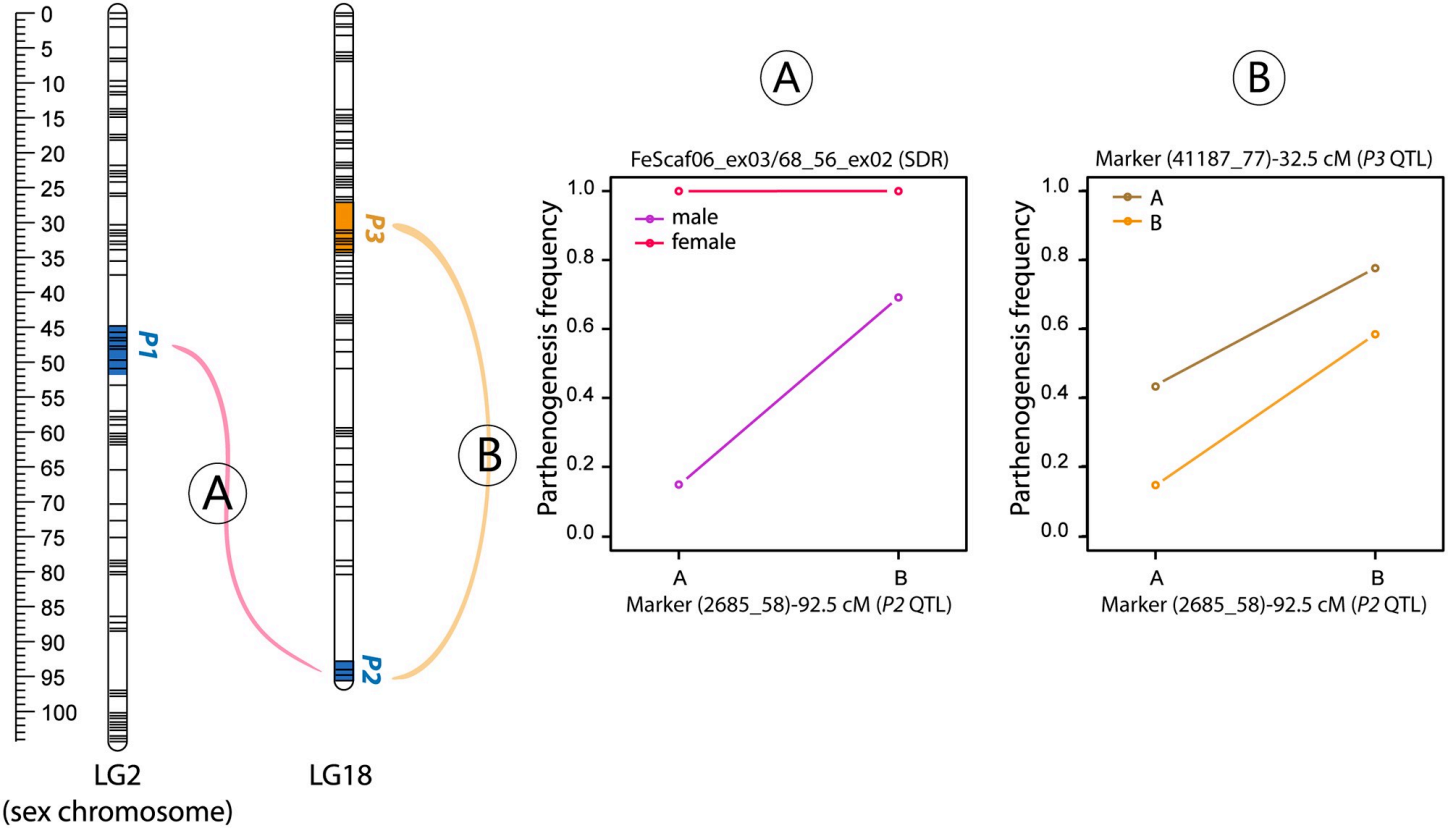
Epistatic interactions between parthenogenetic loci. **A**. Epistatic interaction detected between the sex-determining region (SDR) and the *P2* QTL. The B allele was inherited from the female parent, and the A allele from the male parent. Females can undergo parthenogenesis independently of the allele carried at the *P2* locus whereas males are only parthenogenetic if they carried the B allele at the *P2* locus. **B**. Epistatic interaction between the *P3* and *P2* loci. The combination of B alleles at both *P2* and *P3* loci increases the parthenogenetic frequency. Note that parthenogenesis frequencies were calculated using both males and females.

We observed significant sex-by-genotype interactions for parthenogenetic capacity. The analysis of the full dataset identified an epistatic interaction between the *P2* QTL and the *P1* QTL (Fig 3). When the same analysis was carried out with only the males, this epistatic interaction was not detected (S9 Table). In Fig 3, the B allele was inherited from the female parent, and the A allele from the male parent. All females were parthenogenetic and therefore their parthenogenetic phenotype was independent of the allele carried at the *P2* locus. In contrast, the phenotype of males depended on the allele carried at the *P2* locus.

An additional interaction was detected between the *P2* QTL and the *P3* QTL. In this case, the frequency of P+ individuals was higher when the maternal B allele was present at the *P2* locus and the effect was strongest when the *P3* locus carried the paternal A allele (Fig 3B, S10 Table).

Several additional interactions were detected between the *P2* QTL and markers on several autosomes when the male-only dataset was analysed (S9 Table).

### Identification of candidate genes within the parthenogenesis QTL intervals

A total of 167–169 genes (depending on whether we consider the U or the V, respectively, for *P1*) were identified within the three QTL intervals. Gene Ontology enrichment tools were used to test if some functional categories were over-represented in these genes. BLAST2GO analysis indicated a significant enrichment in processes related to signalling and cell communication (p-value < 0.0001) (S11 Table).

We used several approaches to identify candidate parthenogenesis genes within the three QTL intervals. First, we reasoned that genes involved in parthenogenesis should be expressed in at least one of the gamete types, P+ or P-, where parthenogenesis is initiated. Strains EA1 and RB1 did not produce enough gametes for RNA extraction. We therefore generated RNA-seq data from P+ female and P- male strains from another species within the *E*. *siliculosi* group, *Ectocarpus* species 1 [38] (see methods). We analysed the abundance of the transcripts of orthologs of the 167–169 genes within the three QTL intervals (42/44 genes, 30 genes and 95 genes in the *P1*, *P2* and *P3* intervals respectively, S7 Table). Based on this analysis, 113/119 genes (again, depending on whether we consider the U or the V, respectively) were classed as being expressed in at least one of the gamete types (S12 Table).

Second, we looked for genes that were significantly differentially expressed between P+ and P- gametes, again using the data for *Ectocarpus* species 1 orthologues. Overall, 4902 orthologues were differentially expressed in P+ versus P- strains across the genome, of which 42 corresponded to genes located within the QTL intervals (eight within the *P1*, seven within the *P2* and 27 within the *P3* QTL intervals; Fig 2D, S12 Table). The QTL intervals were therefore significantly enriched in genes that we classed as being differentially expressed between P+ and P- strains (Fisher exact test; p-value = 0.0212).

Third, we looked for polymorphisms with potential effects on the functions of the candidate genes. Comparison of the parental genomic sequences identified a total of 3046 indels and 9702 SNPs within the three QTL intervals, including 46 indels and 152 SNP located in exons (S12 and S13 Tables). In total, 61 genes within the QTL intervals carried SNPs or indels that corresponded to non-synonymous modifications of the coding sequence and were therefore predicted to affect protein function. The male and female SDRs do not recombine [39] and have therefore diverged considerably over evolutionary time. This has included loss and gain of genes but also strong divergence of the genes that have been retained in both regions (gametologs). All SDR genes were therefore retained as candidates (S12 Table).

We then combined the three approaches. The criteria we used were that genes involved in parthenogenesis must be expressed in gametes and they should have either differential expression in P+ versus P- gametes or carry a non-synonymous polymorphism. This reduced the number of candidates to 13/18 (U/V chromosome) genes in the *P1*, nine genes in the *P2* and 50 genes in the *P3* QTL (Fig 2D, S12 Table). Taking genes that were both differentially expressed in P+ versus P- gametes and that carried a non-synonymous polymorphism (S12 Table, Fig 2D) further reduced the list of candidate genes to 8/13 (U/V), one and 15 candidates in *P1*, *P2* and *P3*, respectively.

### Parthenogenetic male gametes exhibit reduced fitness in sexual crosses

It is not clear why some strains of *Ectocarpus* exhibit male gamete parthenogenesis whilst others do not. More specifically, bearing in mind that all strains tested so far exhibit parthenogenesis of female gametes, why are male gametes not parthenogenetic in some lineages? To address this question, we investigated if there were differences in fitness between P- and P+ male gametes for parameters other than parthenogenetic growth. Specifically, we examined fertilisation success (capacity to fuse with a female gamete) and growth of the resulting diploid sporophyte.

**Fig 4:**
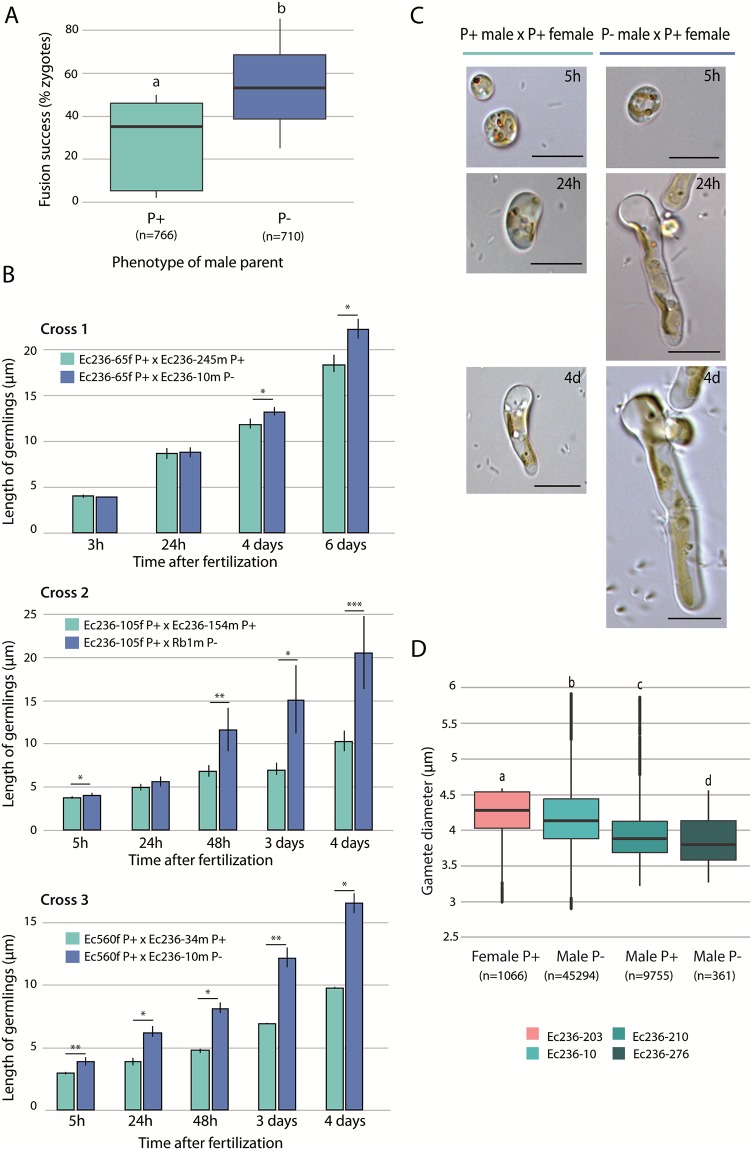
Fitness of parthenogenetic (P+) and non-parthenogenetic (P-) males. **A**. Fertilisation success was assessed by counting the proportion of zygotes obtained after crossing either parthenogenetic (Ec236-34, Ec236-245) or non-parthenogenetic (Ec236-10, Ec236-298) males with parthenogenetic females (Ec236-284, Ec236-39, Ec236-203, Ec560) (n = 1252). Fusion success was significantly higher when the male parent was P- (p = 0.047); significance is represented by different letters). Number of scored germlings is presented in brackets. **B**. Growth of germlings derived from three independent crosses performed between female P+ and male P+ or male P- strains. Significant differences (Wilcox rank sum test) are indicated (*p-value<0.05;** p-value<0.01***p-value<0.001). Between three and fourteen germlings were scored per cross at each time point (see also S13 Table). **C**. Representative images of zygotes at different developmental stages, from a male P- (RB1) x female P+ (Ec236-105) cross and from a male P+ (Ec236-154) x female P+ (Ec236-105) cross. Scale bar = 10 μm. **D**. Sizes of gametes from a parthenogenetic female, a parthenogenetic male and non-parthenogenetic male. The mean diameter of female P+ (Ec236-203, n = 1066), male P+ (Ec236-210, n = 9755) and two P- males (Ec236-276, n = 45294 and Ec236-10 n = 361) lines were measured by cytometry. The values of gamete size shown represent the mean ± s.e. for each individual.

We tested twelve combinations of crosses between four P- or P+ males and five females (S14 and S15 Tables). We fitted a generalized linear mixed model with a logistic regression to the data and observed that the male P- gametes showed a higher rate of fusion with female gametes than the P+ male gametes (p = 0.0048). However, the data showed overdispersion, which needed to be accounted for to avoid biased parameter estimates. Accounting for overdispersion led to a higher but still significant p-value (p = 0.047). Therefore, we concluded that, overall, male P- gametes fuse more efficiently with female gametes than do P+ male gametes. Moreover, embryos arising from P- male gametes also grew significantly more rapidly than embryos derived from fusion with male P+ gametes (Fig 4B and 4C, Mann-Whitney u-test p<0.05, S16 Table).

The overall size of zygotes is expected to be correlated with zygotic and diploid fitness [40–42]. We therefore hypothesised that if P- male gametes are larger, fusion with a female gamete would generate larger (and therefore fitter) zygotes. Measurements of gamete size of P+ and P- strains revealed significant differences in gamete size between different strains (Kruskal-Wallis test, Chi2 = 3452.395, P<2.2e-16, S17 Table, Fig 4D). However, there was no correlation between the parthenogenetic capacity of male gametes and their size, suggesting that the increased fitness of the zygotes was unlikely to be related to the size of the male gametes.

Taken together, these analyses indicate that P+ male gametes exhibit overall reduced fitness in sexual crosses, both in terms of success of fusion with a female gamete and growth of the resulting embryo. We found no link between the size of the male gamete and the capacity to perform parthenogenesis, which excludes the possibility that the fitness decrease is due to the size of the male gamete.

## Discussion

### The genetic architecture of parthenogenesis in *E*. *siliculosus*

In this study, we uncovered the genetic architecture of parthenogenesis in the brown alga *E*. *siliculosus* and demonstrated that this trait is controlled by two major and one minor QTL loci that, together, account for 44.8% of the phenotypic variation. The two main QTL loci were located at the SDR of the sex chromosome and on LG18, respectively, and the minor QTL was also located on LG18. Analysis of differential expression patterns and polymorphism for genes within the QTL intervals allowed the establishment of a list of 77 candidate parthenogenesis genes: 13/18 genes within the sex chromosome QTL interval (in the U and V respectively), nine genes within the *P2* locus and 50 within the interval of the minor *P3* locus. Interestingly, within the major *P2* QTL a strong candidate gene coded for a membrane-localized ankyrin repeat-domain palmitoyltransferase (Ec-20_004890). In *S*. *cerevisiae*, genes belonging to the same family are involved in the gamete pheromone response pathway, regulating the switching between vegetative and mating states [43,44].

### A key role for the sex chromosome in parthenogenesis

Our results reveal a critical role for the sex chromosome in the control of parthenogenesis, with a major effect QTL being located within the SDR. In females, parthenogenesis occurs regardless of the alleles carried at the *P2* and *P3* loci, whereas males are parthenogenetic only when they have a P+ allele at least at one of the autosomal QTL. Accordingly, epistatic interactions between the SDR and the major *P2* QTL locus were detected only when both males and females were included in the analysis. The epistatic effect could be due to the production of a repressor of parthenogenesis by the male V-specific region or to the production of an activator of parthenogenesis by the female U-specific region (in either case the activator or repressor could be directly encoded by the SDR or produced indirectly as part of the male or female sex-differentiation programs). Interestingly, several genetically male kelp strains of *Laminaria pallida* and *Macrocystis pyrifera* were shown to produce unusual reproductive structures resembling eggs, which were capable of parthenogenesis (Ingo Maier, D. Muller, pers. commun.). These observations suggest that, if an activator of parthenogenesis is produced in the female, then this probably occurs indirectly as part of the female sex-differentiation program rather than being directly encoded by the SDR, at least in these species (because these kelp individuals lacked a U sex chromosome).

### Male fitness effects of parthenogenetic capacity

Our results indicate that parthenogenetic capacity has a negative impact on male gamete fitness during sexual reproduction. Zygotes produced by fusion with P- male gametes grew significantly faster than those produced by fusion with P+ male gametes. We also observed that P- male gametes exhibited a higher percentage of successful fertilisations with female gametes than P+ male gametes. The underlying mechanistic basis for the decreased fitness of P+ compared with P- male gametes is currently elusive. P+ and P- alleles may have a direct phenotypic effect on the male gametes, although we have not detected any difference in parameters such as gamete size. A link between mitochondria metabolism and parthenogenetic capacity has been evoked in another brown algal species [25].

Moreover, parthenogenesis-promoting alleles at *P2* and *P3* may have an indirect effect on fitness because they favour asexual rather than sexual reproduction, leading to reduced recombination rates and hence reduced capacity to eliminate deleterious mutations due to Hill-Robertson interference [45,46]. It is not clear, however, whether the degree of asexual reproduction in field populations would be sufficient for this effect to be significant and note also that increased parthenogenesis is also expected to lead to an increase in haploid purifying selection, which would tend to counteract the effect of reduced recombination to some extent.

### Maintenance of polymorphisms at the parthenogenesis QTLs

Although we have not investigated allele frequencies in natural populations in this study, an earlier analysis of the Naples *E*. *siliculosus* population indicated that about 12.5% of male individuals were phenotypically P+ [47], suggesting that P+ alleles of parthenogenesis QTLs may be relatively abundant in this population. If P+ males are expected to exhibit reduced fitness in sexually reproducing populations, and females are phenotypically P+ regardless of the allele at the *P2* and *P3* QTL, how can autosomal P+ alleles be preserved in the population? In other words, how is polymorphism maintained at the parthenogenesis QTLs? In the following paragraphs, we discuss several hypotheses that may explain the maintenance of this polymorphism. Note that it is likely that different evolutionary forces have been acting on the sex chromosome QTL (*P1*) compared to the autosomal QTLs (*P2* and *P3*) and these differences are discussed where appropriate.

One interesting possibility is that parthenogenesis is a sexually antagonistic trait (or at least differentially selected in males versus females). Indeed, this is probably the most likely explanation for the existence of the fixed polymorphism between males and females at the *P1* locus (SDR QTL) if we consider that parthenogenetic capacity will tend to be beneficial for females but less beneficial for males because of negative effects on male sexual reproduction. The incidence of parthenogenesis in males and females would reflect the costs and benefits of each mode of reproduction in each of the sexes. Parthenogenesis in males may be more costly because of potential mutations caused by reactive oxygen species production during active swimming [48], which would be exposed during (haploid) parthenogenetic development. Moreover, the smaller size of male gametes may impede early development (for example due to limited resources to invest in parthenogenetic germination). In females, whose gametes have a passive behaviour and are bigger, a ‘facultative’ parthenogenesis strategy could be favoured, specifically in conditions of sperm limitation. Although we could not measure the effect of parthenogenetic capacity on female gamete fitness, because all females were phenotypically P+, sexual antagonism would be consistent with the pervasiveness of the female P+ phenotype and the differences in fitness between P+ and P- males. This phenomenon would be particularly relevant in spatially heterogeneous and/or unpredictable environments, where the P+ or P- allele(s) in males would be alternatively selected for or against, depending on female density.

Another potential mechanism for the maintenance of genetic variation is opposing selection during the diploid and haploid stages of biphasic life cycles, also known as ploidally-antagonistic selection [26]. Parthenogenesis could be considered an example of a trait under ploidally/generation antagonistic selection because P- alleles transmitted by the male gamete are advantageous to the diploid (sporophyte) generation (because zygotes grow faster if the father is P-) but detrimental to the haploid (partheno-sporophyte) generation (because if they do not find a female gamete, male gametes that carry a P- allele die). Ploidally-antagonistic selection has been proposed to have a significant impact on major evolutionary dynamics, including the maintenance of genetic variation and the rate of adaptation [26,49,50]. Note, however, that the detrimental effect on males will depend on the availability of female gametes so that, under conditions where sexual reproduction is favoured, P- alleles should be beneficial for males and P+ detrimental. Mathematical modelling [51] predicts that when selection differs between the sexes (and in particular when the gametophyte-deleterious allele is neutral or slightly beneficial in one of the sexes), being close to or within the SDR expands the range of parameters allowing generation-antagonistic mutations to spread. Indeed, conflict arising from generation-antagonism or from differences in selection in gametophytes compared with the sporophyte generation is best resolved by complete linkage to the SDR [51]. The effect of ploidally-antagonistic selection is therefore likely to be strongest for the *P1* locus but this effect could also influence allele frequencies at the *P2* and *P3* loci.

Temporal or spatial changes in population density are extremely common and these are expected to cause strong fluctuating selection on life-history traits, thereby contributing to the maintenance of genetic polymorphism in populations (e.g. [28]). Polymorphism can also be maintained when selection fluctuates as a result of developmental changes, for example when selection only occurs in one sex, resulting in the sheltering of alleles in the sex where they are not under selection [52]. In the case of *E*. *siliculosus*, for example, autosomal parthenogenesis alleles could be protected from selection because they have no phenotypic effect in females. In other words, if phenotypic expression of autosomal P- allele(s) is limited to males, fluctuating selection of this sex-limited trait could lead to the existence of a protected polymorphism, and contribute to the maintenance of genetic variance at the autosomal QTLs. The P+ allele would be maintained because it is advantageous in males when females are rare or when populations have low density. Note that this mechanism is not relevant for the *P1* locus because alleles are systematically transmitted to individuals of the same sex.

### Is parthenogenesis adaptive?

In the brown algae, the ancestral state appears to have been sexual reproduction through fusion of strongly dimorphic gametes (oogamy), that were incapable of parthenogenesis ([53], reviewed in [29]). Gamete parthenogenesis evolved secondarily, and a challenge for understanding the adaptive nature of gamete parthenogenesis in these organisms would be to identify the conditions under which it occurs in nature. Brown algae exhibit a remarkable degree of reproductive plasticity during their life cycle [19,54] and it is possible that this plasticity is related to their capacity to adapt to new conditions, in particular low population densities or very fragmented habitats where finding a partner may be problematic. Our data supports the idea that P+ individuals may employ a bet-hedging strategy in the sense that their gametes are capable of reproducing either sexually or parthenogenically, thereby ensuring reproduction in heterogeneous and unpredictable environments.

It has been predicted that in marginal populations, or other situations where mates are limited, parthenogenesis could be adaptive and thus selectively favoured [55]. In animals (fish, *Drosophila*) rapid transition between reproductive strategies were observed following the removal of the mate, supporting the hypothesis that parthenogenesis has a reproductive advantage under conditions of isolation from potential mates [56]. A recent study of *Ectocarpus siliculosus* populations in NW of France has shown that asexual populations are prevalent in the field, but gamete parthenogenesis does not appear to play a critical role in this population, and instead, sporophytes are produced mainly from the development of diploid, asexual spores [57]. However, parthenogenesis is an important process in field populations of other brown algal species, such as *Scytosiphon lomentaria* [20]. Additional population data are required, specifically parthenogenesis allele frequencies for natural populations where individuals are found at different densities, for marginal versus central populations and for different types of habitat, to further investigate whether there is an adaptive benefit to parthenogenesis.

## Material and methods

### *E*. *siliculosus* cultures

Gametophytes of *E*. *siliculosus* (S1 Table) were maintained in culture as previously described [58]. *E*. *siliculosus* strains can be maintained in the gametophyte generation indefinitely, with weekly changes in culture media [58]. Clonal cultures of male and female gametophytes were subjected to strong light (100 μm photons/m^2^/s) and low temperatures (10°C) to induce fertility resulting in the release of large numbers of gametes (>10e^5^). Gametes were allowed to settle on coverslips and their development was monitored under an inverted microscope (Olympus BX50).

### Evaluation of parthenogenetic capacity and sex

The sex of the gametophytes was assessed using PCR-based sex markers (FeScaf06_ex03 and 68_56_ex02, which correspond to conserved regions within the U and V SDRs, respectively, [39,59]). Parthenogenetic capacity was evaluated after 15 days in culture using an inverted microscope (Olympus CKX41). Strains were scored as parthenogenetic if more than 4% of parthenotes grew beyond the ten-cell stage.

### Cross design, culturing and phenotyping

A cross between a parthenogenetic female (strain EA1) and a non-parthenogenetic male (strain RB1) was carried out using a standard genetic cross protocols [60] and a diploid heterozygous sporophyte was isolated (Ec236) (Fig 1; S1 Table). At maturity, the sporophyte (strain Ec236) produced unilocular sporangia, i.e, the reproductive structure where meiosis takes place (Fig 1). A total of 272 unilocular sporangia were isolated, and one gametophyte was isolated from each.

The 272 strains of the EA1 x RB1 derived segregating population were cultivated in autoclaved sea water supplemented with half strength Provasoli solution [61] at 13°C, with a light dark cycle of 12:12 (20 μmol photon m^-2^ s^-1^) using daylight-type fluorescent tubes [58]. All manipulations were performed in a laminar flow hood under sterile conditions. We phenotyped the strains for parthenogenetic capacity (P+ or P-) and for sex (male or female). Parthenogenetic capacity was was evaluated after 15 days in culture. Parthenogenesis in *E*. *siliculosus* is a binary trait. Strains were scored as parthenogenetic (P+) if more than 4% of parthenotes grew beyond the ten-cell stage. In order to assess phenotype stability, gametophytes were sub-cultivated in different conditions for two weeks and then exposed to high intensity light to induce fertility. Parthenogenetic capacity was measured using the released gametes (S3 Table). To test the stability of the phenotype across generations, we cultivated partheno-sporophytes and induced them to produce unilocular sporangia and release meio-spores to obtain a new generation of gametophytes. The parthenogenetic capacity of gametes derived from these second-generation gametophytes was then tested (S3 Table). Note that this experiment is feasible in P- males because a very small proportion (less than 4%) of their gametes are nevertheless able to develop into mature partheno-sporophytes.

Each of the 272 gametophytes of the EA1 x RB1 segregating family was frozen in liquid nitrogen in a well of a 96 well plate. After lyophilization, tissues were disrupted by grinding. DNA of each gametophyte was extracted using the NucleoSpin Plant II kit (Macherey-Nagel, Germany) according to the manufacturer’s instructions and stored at -80°C. Sexing of gametophytes was carried out using two PCR-based sex markers, one for each sex (FeScaf06_ex03 forward: CGTGGTGGACTCATTGACTG; FeScaf06_ex03 reverse: AGCAGGAACATGTCCCAAAC; 68_56_ex02 forward: GGAACACCCTGCTGGAAC; 68_56_ex02 reverse: CGCTTTGCGCTGCTCTAT) [39]. PCR was performed with the following reaction temperatures: 94°C 2min; 30 cycles of 94°C 40s, 60°C 40s and 72°C 40s; 72°C 5min, and with the following PCR mixture 2 μL DNA, 100 nM of each primers, 200 μM of dNTP mix, 1X of Go Taq green buffer, 2 mM of MgCl2, 0.2 μL of powdered milk at 10% and 0.5 U of Taq polymerase (Promega).

### DNA extraction and library RAD sequencing

A double digest RAD sequencing (ddRAD-seq) library was generated using 152 individuals from the EA1 x RB1 segregating population. Parthenogenetic individuals were selected (37 females and 36 males) as well as non-parthenogenetic males (79 individuals). DNA extraction was performed for each individual (Macherey-Nagel, NucleoSpin Plant II kit (GmbH & Co.KG, Germany) and DNA quantity was measured and standardized at 100 ng using a PicoGreen (Fischer Scientific) method for quantification. The DNA quality was checked on agarose gels.

The ddRAD-seq library was constructed as in [62] using *Hha*I and *Sph*I restriction enzymes (New England Biolabs, https://www.neb.com/). Those enzymes were selected based on an *in silico* digestion simulation of the Ec32 reference genome [18] using the R package SimRAD [63]. After digestion, samples were individually barcoded using unique adapters by ligation with T4 DNA ligase (New England Biolabs, https://www.neb.com/). Then, samples were cleaned with AMPure XP beads (Beckman Coulter Genomics), and PCR was performed with the Q5 hot Start High-Fidelity DNA polymerase kit (New England Biolabs, https://www.neb.com/) to increase the amount of DNA available for each individual and to add Illumina flowcell annealing sequences, multiplexing indices and sequencing primer annealing regions. After pooling the barcoded and indexed samples, PCR products of between 550 and 800 bp were selected using a Pippin-Prep kit (Sage Science, Beverly, MA, USA), and the library was quantified using both an Agilent 2100 Bioanalyzer (Agilent Technologies) and qPCR. The library was sequenced on two Illumina HiSeq 2500 lanes (Rapid Run Mode) by UMR 8199 LIGAN-PM Genomics platform (Lille, France), with paired-end 250 bp reads.

### Quality filtering and reference mapping

The ddRAD-seq sequencing data was analysed with the Stacks pipeline (version 1.44, [31]). The raw sequence reads were filtered by removing reads lacking barcodes and restriction enzyme sites. Sequence quality was checked using a sliding window of 25% of the length of a read and reads with <90% base call accuracy were discarded. Using the program PEAR (version 0.9.10, [64]) paired-end sequencing of short fragments generating overlapping reads were identified and treated to build single consensus sequences. These single consensus sequences were added to the singleton rem1 and rem2 sequences produced by Stacks forming a unique group of singleton sequences. For this study, paired-end reads and singleton sequences were then trimmed to 100 bp with the program TRIMMOMATIC [65]. The genome of the male parent of the population (strain RB1) was recently sequenced to generate an assembly [66] guided by the *Ectocarpus* species 7 reference genome published in 2010 [67]. We performed a *de novo* analysis running the denovo_map.pl program of Stacks. Firstly, this program assembles loci in each individual *de novo* and calls SNPs in each assembled locus. In a second step, the program builds a catalog with the parental loci and in a third step, loci from each individual are matched against the catalogue to determine the allelic state at each locus in each individual. We then used BWA (Li, H. Aligning sequence reads, clone sequences and assembly contigs with BWA-MEM.arXiv:1303.3997) to align the consensus sequence of the catalog loci to the reference genome and used the Python script “integrate_alignments.py” of the Stacks pipeline to integrate alignment information back into the original de novo map output files [68]. In a final step, SNPs were re-called for all individuals at every locus and exported as a vcf file.

### Genetic map construction and QTL mapping

The vcf file obtained with the Stacks pipeline was first filtered to keep only loci with maximum of 10% of missing samples and samples with a maximum of 30% of missing data. The program Lep-MAP3 (LP3) [69] was used to construct the genetic map (S18 Table). LP3 is suitable to analyse low-coverage datasets and its algorithm reduces data filtering and curation on the data, yielding more markers in the final maps with less manual work. In order to obtain the expected AxB segregation type for this haploid population, the pedigree file was constructed by setting the parents as haploid grand-parents and two dummy individuals were introduced for parents. The module ParentCall2 of LP3 took as input the pedigree and the vcf files to call parental genotypes. The module SeparateChromosomes2 used the genotype call file to assign markers into linkage groups (LGs). Several LOD score limits were tested to obtain an optimal LOD score of 8 giving a stable number of LGs. The module JoinSingles2All was then run to assign singular markers to existing LGs by computing LOD scores between each single marker and markers from the existing LGs. The module OrderMarkers2 then ordered the markers within each LG by maximizing the likelihood of the data given the order. Sex averaged map distances were computed and 10 runs were performed to select the best order for each LG, based on the best likelihood. This module was run with the parameters grandparentPhase = 1 and outputPhasedData = 1 in order to obtain phased data for QTL mapping. This phased data was converted to fully informative genotypic data using the script map2gentypes.awk distributed with the LP3 program. The PCR-based sex markers (FeScaf06_ex03 and 68_56_ex02, which correspond to conserved regions within the U and V SDRs, respectively) were recoded as a unique sex marker, with the B allele for females and A allele for males.

Identification and mapping of QTLs were carried out using R/qtl (version 1.39–5) and MapQTL version 5. Non-parametrical statistics were used because parthenogenetic capacity was phenotyped as a binary trait (either 0 for non-parthenogenetic or 1 for parthenogenetic). In R/qtl, the scanone function was used with the “binary” model to perform non-parametrical interval mapping with the binary or Haley-Knott regression methods. The function refineqtl was used to obtain improved estimates of the locations of the QTLs. In MapQTL, the Kruskal-Wallis non-parametric method was used. To determine the statistical significance of the major QTL signal, the LOD significant threshold was determined by permutation.

### Transcriptome data

The small number of gametes released from *Ectocarpus siliculosus* strains did not allow RNA-seq data to be obtained from this species. To analyse gene expression in P- (male) and P+ (female) gametes, we therefore used two *Ectocarpus* species 1 strains belonging to the same *Ectocarpus siliculosi* group [33], a P- male (NZKU1_3) and a P+ female (NZKU32-22-21), which produce sufficient numbers of gametes for RNA extraction. Note that, although there are currently only three official species in the *Ectocarpus* genus, genetic analysis has shown that there are at least 15 species in the genus with most differences being cryptic [33].

Gametes of male and female *Ectocarpus* species 1 were concentrated after brief centrifugation, flash frozen and stored at -80°C until RNA extraction. RNA was extracted from duplicate samples using the Qiagen RNeasy plant mini kit (www.qiagen.com) with an on-column DNase I treatment. Between 69 and 80 million sequence reads were generated for each sample using Illumina HiSeq 2000 paired-end technology with a read length of 125 bp (Fasteris, Switzerland) (S19 Table). Read quality was assessed with FastQC (http://www.bioinformatics.babraham.ac.uk/projects/fastqc), and low quality bases and adapter sequences were trimmed using Trimmomatic (leading and trailing bases with quality below 3 and the first 12 bases were removed, minimum read length 50bp) [65]. High score reads were used for transcriptome assembly generated with the Trinity *de novo* assembler [70] with default parameters and normalized mode. RNA-seq reads were mapped to the assembled reference transcriptome using the Bowtie2 aligner [71] and the counts of mapped reads were obtained with HTSeq [72]. Expression values were represented as TPM and TPM<1 was applied as a filter to remove noise if both replicates of both samples exhibit it. Differential expression was analysed using the DESeq2 package (Bioconductor; [73]) using an adjusted p-value cut-off of 0.05 and a minimal fold-change of two. The reference transcripts were blasted to the reference genome Ec32 predicted proteins (http://bioinformatics.psb.ugent.be/orcae/overview/EctsiV2) (e-value cut-off = 10e^-5^) and the orthology relationship between *Ectocarpus* species 1 and Ec32 (*Ectocarpus* species 7) was established based on the best reciprocal blast hits.

### Identification of candidate genes in the QTL intervals

We used two methods to identify putative candidate genes located in the QTL intervals. First, a marker-by-marker method, by mapping the sequences of the markers located within each QTL interval to the reference genome of the closely related reference species strain Ec32 (Cock et al., 2010). When a sequence successfully mapped to the Ec32 genome, a coordinate was recorded for the marker, relative to its position on the physical map of Ec32. The second method used the same approach but was based on the reference genome of the paternal strain of the population (strain RB1). There were some differences between the two lists obtained by the two methods, which are due to the following factors: (a) because the assembly of the RB1 genome was guided by the Ec32 reference genome and its annotation was based on Ec32 transcriptomic data, the RB1 genome potentially lacks some genes that would be due to loci such as genes that are unique to the species *E*. *siliculosus* (RB1 strain) being omitted during the guided assembly. Hence the list obtained with the first method (using the Ec32 genome) contains genes that are absent from the RB1 genome; (b) while the two species are closely related, they are not identical, and the *E*. *siliculosus* genetic map exhibited some rearrangements compared to Ec32 which placed some markers, along with associated genes, into the QTL intervals (these missing markers were located elsewhere on the Ec32 genome). In summary, the list obtained with Ec32 genome contained some genes that are missing from the RB1 genome because of its imperfect guided assembly and the list obtained with the RB1 genome contained some genes absent from the corresponding intervals on Ec32 because of rearrangements. A final, conservative list of candidate genes was obtained by merging the two lists in order not to omit any gene that were potentially located within the intervals.

### SNP and indel detection method

Draft genomes sequences are available for the parent strains RB1 and EA1 [66]. Using Bowtie2, we aligned the EA1 genome against the RB1 genome and generated an index with sorted positions. The program samtools mpileup [74] was used to extract the QTL intervals and call variants between the two genomes. The positions of variants between the two genomes were identified and filtered based on mapping and sequence quality using bcftools [75]. The annotation file generated for the RB1 genome was then used to select SNPs and indels located in exons of protein-coding genes for further study (bcftool closest command). The effect of polymorphism on modification of protein products was assessed manually using GenomeView [76], the RB1 genome annotation file (gff3) and the vcf file for each QTL region.

### GO term enrichment analysis

A Gene Ontology enrichment analysis was performed using two lists of genes: a predefined list that corresponded to genes from all three QTL intervals and a reference list including all putative genes in the mapped scaffolds based on the Ec32 reference genome and that had a GO term annotation. The analysis was carried out with the package TopGO for R software (Adrian Alexa, Jörg Rahnenführer, 2016, version 2.24.0) by comparing the two lists using a Fisher’s exact test based on gene counts.

### Epistasis analysis

Epistasis analysis was carried out with the R package R/qtl (version 3.3.1). Two analyses were performed, one with the full data set (female and male genotypes generated with RAD-seq method) and the second with only the male individuals. For both analyses, the scantwo function from R/qtl were used with the model “binary” as the phenotypes of the individuals is either 1 (P+) or 0 (P-).

### Fitness measurements

Reproductive success was assessed in the segregating population by measuring the capacity of male P+ and P- gametes to fuse with female gametes and by measuring the length of the germinating sporophytes derived from these crosses. For this, we crossed males and females as described in [60]. Briefly, we mixed the same amount of male and female gametes (app. 1x10^3^ gametes) in a suspending drop, and the proportion of gametes that succeeded in fusing was scored. Two different P+ males (Ec236-34 and Ec236-245) and two different P- males (Ec236-10 and Ec236-298) were crossed with five different females (Ec236-39; -203; -65; -284 and Ec560) (S14 Table). Between 18 and 254 germlings were counted for each cross. To determine whether there was a difference between the capacity of P+ and P- male gametes to fuse with female gametes, we applied a generalized linear mixed model with a logistic regression (family = binomial) using the glmer function from the R package lme4 (details in S14 Table). The model was run with and without observation-level random effects to account for overdispersion [77].

The length of zygotes derived from three different crosses between female strains and either male P- or male P+ strains were measured after between 3h to 4 days of development using Image J 1.46r [78]. For all datasets, the assumption of normality (Shapiro test) and the homoscedasticity (Bartlett’s test) were checked. The latter’s assumptions were not met for zygote length, and consequently statistical significance differences at each time of development was tested with a non-parametrical test (Mann Whitney U-test, α = 5%).

### Measurement of gamete size

Gamete size was measured for representative strains of each parthenogenetic phenotype found in the segregating population (P+ and P-) (S3 Table). Synchronous release of gametes was induced by transferring each gametophyte to a humid chamber in the dark for approximately 14 hours at 13°C followed by the addition of fresh culture medium under strong light irradiation. Gametes were concentrated by phototaxis using unidirectional light, and collected in Eppendorf tubes. Gamete size was measured by impedance-based flow cytometry (Cell Lab QuantaTM SC MPL, Beckman Coulter). A Kruskal-Wallis test (α = 5%) followed by a posthoc Dunn’s test for pairwise comparisons were performed using R software to compare female and male gamete size (S16 Table).

## Supporting information

S1 Fig Pedigree of the strains used in this study indicating all the crosses performed. Male strains are represented in green, female strains in dark red. P+, positive parthenogenetic capacity; P-, negative parthenogenetic capacity.
(TIF)

S1 Table Summary of the strains used in this study. SP: sporophyte; GA: gametophyte; P+: positive parthenogenetic capacity; P- negative parthenogenetic capacity.
(XLSX)

S2 Table Contingency table for parthenogenetic capacity and sex. P+: positive parthenogenetic capacity; P- negative parthenogenetic capacity.
(XLSX)

S3 Table Parthenogenetic capacity and sex of the 272 individuals of the segregating population. Strains used for the RAD-seq, gamete size measurements and fitness measurements are marked with a cross. P+, positive parthenogenetic capacity; P-, negative parthenogenetic capacity.
(XLSX)

S4 Table Summary of the phenotyping and sexing of strains grown under different culture conditions and after several generations. P+, positive parthenogenetic capacity; P-, negative parthenogenetic capacity.
(XLSX)

S5 Table Phenotypes of the progeny derived from two different heterozygous sporophytes obtained by crossing a male P+ strain and a female P+ strain. (XLSX)

S6 Table Statistics for the genetic map. (XLSX)

S7 Table QTL analysis results. For each QTL, the name, the linkage disequilibrium (LD) within the chromosome, the significance obtained with the Kruskal-Wallis test and the percentage of variance explained (PVE) determined using the Interval Mapping method (IM) is given. The number of genes found in each QTL interval is also indicated.
(XLSX)

S8 Table Genotypes of strains used for the cross between two parthenogenetic strains. P+, positive parthenogenetic capacity; P-, negative parthenogenetic capacity. Allele A represents the paternal allele, allele B the maternal allele.
(XLSX)

S9 Table Epistatic interactions detected for parthenogenesis loci using the full dataset (male and female individuals genotyped with the ddRAD-seq method, first table) and using a subset with only male individuals (second table). The column "interaction" indicates the chromosomal locations of the pairs of loci that were found to interact, with "Pos1f" and "Pos2f" referring to the estimated positions of the QTL in cM. "Lod.full" indicates the improvement in the fit of the full 2-locus model over the null model. This measurement indicates evidence for at least one QTL, allowing for interaction. "Lod.fv1" measures the increase when the full model with QTLs on chromosomes j and k is compared to a single QTL on either chromosome j or k. This measurement indicates evidence for a second QTL allowing for the possibility of epistasis. "Lod int" measures the improvement in the fit of the full model over that of the additive model and so indicates evidence for interaction. "Pos1a" and "pos2a" are the estimated positions (in cM) of the QTL under the additive model. "Lod.add" measures the improvement comparing with the additive model. This measurement indicates evidence for at least one QTL assuming no interaction. "Lod.av1" measures the increase when the additive model with QTLs on chromosomes j and k is compared to the single QTL model with a single QTL on chromosome j and k. This measurement indicates evidence for a second QTL assuming no epistasis.
(XLSX)

S10 Table Parthenogenetic phenotypes of male and female progeny of the cross between strains EA1 and RB1 in relation to their genotypes at the P2 and P3 parthenogenesis QTLs. P+, positive parthenogenetic capacity. Allele A represents the paternal allele, allele B the maternal allele.
(XLSX)

S11 Table List of the top 20 GO terms identified in the gene ontology analysis (TopGO) for genes located within the QTL intervals. (XLSX)

S12 Table Abundances of transcripts of *Ectocarpus* species 1 orthologs of genes in the *E*. *siliculosus* parthenogenesis QTL intervals. Levels of gene expression are represented as transcript per million, TPM for P- (male) versus P+ (female) gametes. Information about the type of polymorphism in the parental strains of the *E*. *siliculosus* segregating population (EA1 female and RB1 male) is also included. Genes represented in Fig 2 are highlighted in bold. "-" means that there was no best reciprocal ortholog with a detectable level of expression in *Ectocarpus* species 1. Pseudogenes in the sex-determining region were removed except for those which are part of a gametologue pair, which are italicized.
(XLSX)

S13 Table List of polymorphisms in coding sequence of genes located within the three parthenogenesis QTL intervals. (XLSX)

S14 Table Fusion success of male P- and P+ gametes with female gametes. The total number of individuals corresponds to the total number of scored individuals (developing either by parthenogenesis or derived from fusion of gametes).
(XLSX)

S15 Table Genotypes of strains used for measurement of fitness. P+, positive parthenogenetic capacity; P-, negative parthenogenetic capacity. Allele A represents the paternal allele, allele B the maternal allele.
(XLSX)

S16 Table Results of the statistical test performed to study growth of zygotes from crosses using male strains with different parthenogenetic capacity. (XLSX)

S17 Table Pairwise statistical tests carried out to determine whether there were significant size differences between P+ female, P+ male and P- male gametes. Two P- male strains were used (Ec236-10 and Ec236-276), one P+ female (Ec236-203) and one P+ male (Ec236-210). A Kruskal-Wallis test indicated significant difference in gamete size. A posthoc Dunn's test revealed, by pairwise comparison of groups, that the sizes of gametes in each group (female P+, male P+ and males P-) were significantly different.
(XLSX)

S18 Table Genotype data used for the construction of the *E*. *siliculosus* genetic map. Sex is coded as 0 for females and 1 for males. Parthenogenesis is coded as 0 for P- and 1 for P+. The id corresponds to the progeny reference number. Linkage groups are listed in the second column and genetic distances in the third.
(XLSX)

S19 Table Summary of the sequencing methods used and the data obtained. (XLSX)
