# Supplementary figures and images for: A key role for sex chromosomes in the regulation of parthenogenesis in the brown alga Ectocarpus
Source: PLoS Genet. 2019 Jun 13;15(6):e1008211. doi: 10.1371/journal.pgen.1008211 (PMC6592573; doi:10.1371/journal.pgen.1008211)

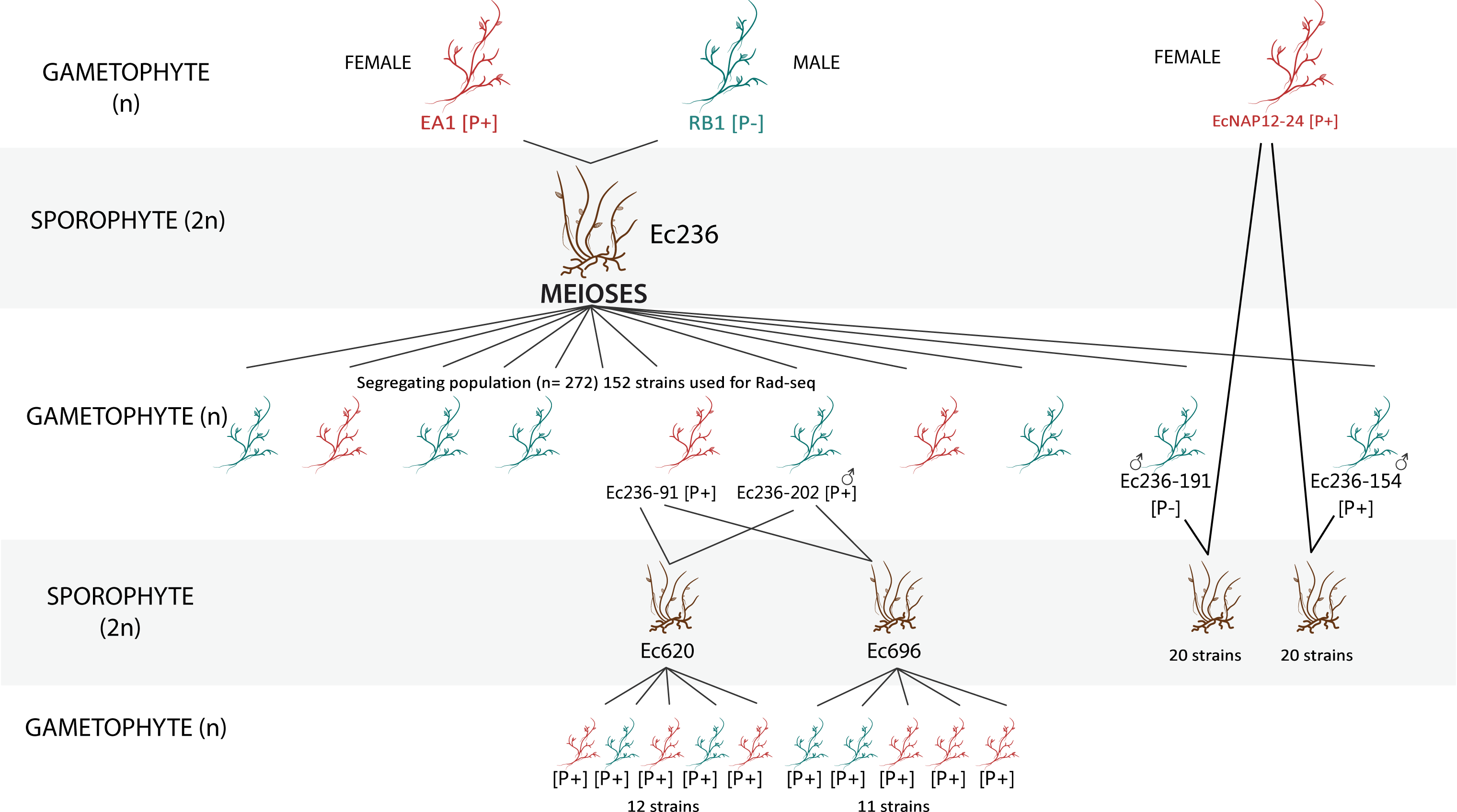

Supplement: S1 Fig — Male strains are represented in green, female strains in dark red. P+, positive parthenogenetic capacity; P-, negative parthenogenetic capacity. (TIF) [file pgen.1008211.s001.tif]
